# Antarctic soil prokaryotic diversity: a dataset of 319 metagenome-assembled genomes from Deception and Livingston Islands

**DOI:** 10.1128/mra.01346-25

**Published:** 2026-02-18

**Authors:** William B. Medeiros, Victor B. Centurion, Jéssica B. Silva, Alysson W.F. Duarte, Kelly J. Hidalgo-Martinez, Juliana A. dos Santos, Daniel D. P. S. Penna, Caner Bagci, Nadine Ziemert, Valéria M. Oliveira

**Affiliations:** 1 Microbial Resources Division, Research Center for Chemistry, Biology, and Agriculture (CPQBA), Universidade Estadual de Campinas (UNICAMP) 28132, Paulínia, São Paulo, Brazil; 2 Biology Institute, Universidade Estadual de Campinas – UNICAMP 28132, Campinas, São Paulo, Brazil; 3 Laboratory of Microbiology, Immunology, and Parasitology, Federal University of Alagoas 28112, Arapiraca, Alagoas, Brazil; 4 Interfaculty Institute of Microbiology, and Infection Medicine Institute for Bioinformatics and Medical Informatics, German Centre for Infection Research (DZIF) 459706, Tübingen, Germany; DOE Joint Genome Institute, Berkeley, California, USA

**Keywords:** Antarctic metagenomics, prokaryotic diversity, extreme environments

## Abstract

A total of 319 bacterial metagenome-assembled genomes (MAGs) were recovered from soil samples collected on the Antarctic Peninsula (Deception and Livingston Islands). These MAGs reveal microbial life’s phylogenetic diversity and functional potential in extreme polar environments, providing resources for advancing microbial ecology, evolution, and Antarctic biotechnology.

## ANNOUNCEMENT

**Fig 1:**
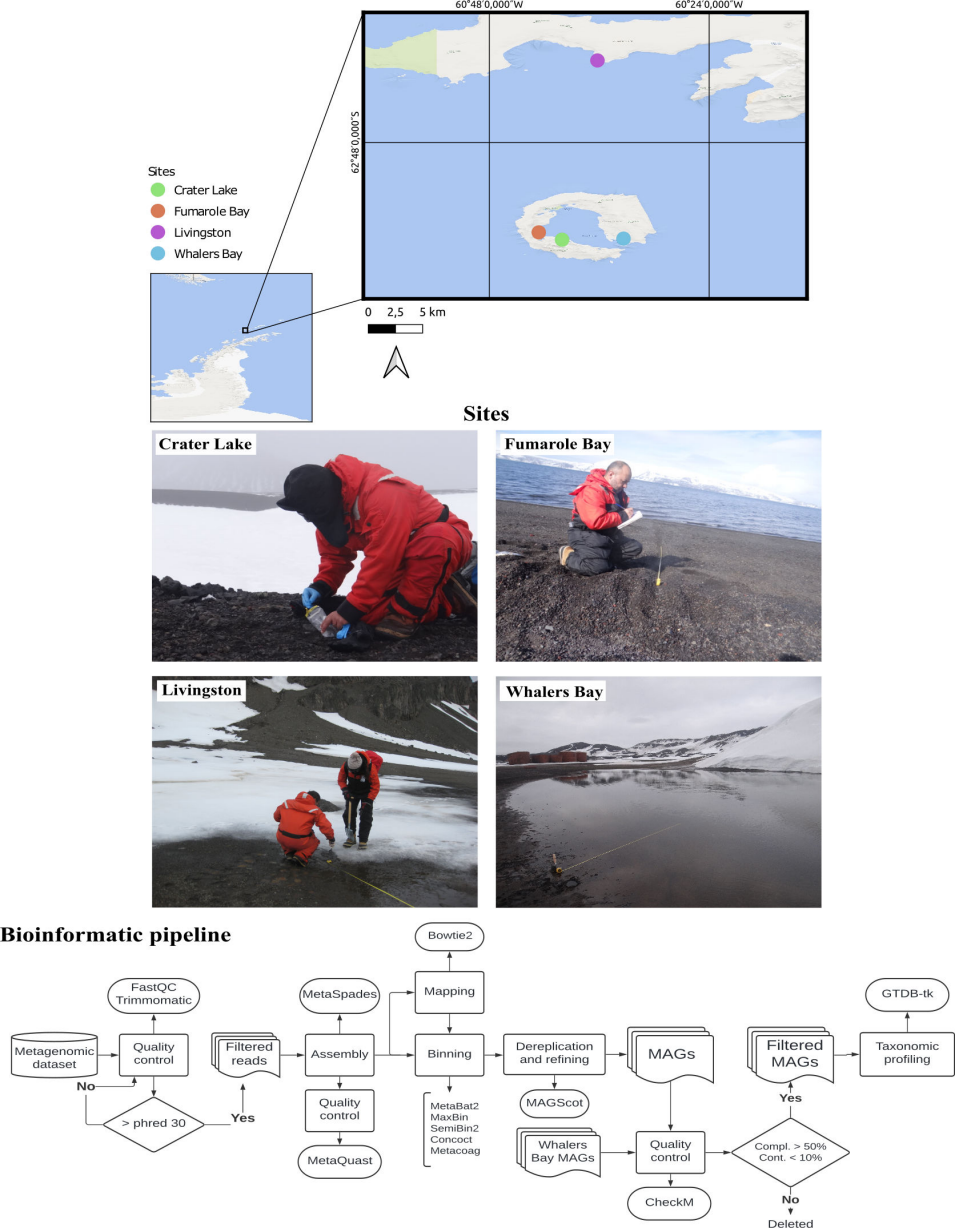
Sampling site map and bioinformatic workflow. The top panel is a map of the Antarctic Peninsula showing sampling sites on Deception Island (Crater Lake [green], Fumarole Bay [orange], and Whalers Bay [blue]) and Livingston Island (Hannah Point [purple]). Insets show fieldwork conducted at each site: Crater Lake (top left panel), Fumarole Bay (top right panel), Livingston (bottom left panel), and Whalers Bay (bottom right panel). The bottom panel provides an overview of the bioinformatic pipeline for recovering and analyzing MAGs from metagenomic data sets. Squares indicate processing steps, and rounded rectangles (ovals) represent software or tools used.

Metagenome-assembled genomes (MAGs) are key for exploring Antarctic microbial diversity, revealing the genetic and metabolic potential of uncultured microorganisms and their adaptations across terrestrial and marine habitats. Antarctic MAG studies have uncovered novel taxa (1) and distinct metabolic pathways for xenobiotic degradation and survival under low temperature and nutrient scarcity (2, 3). We retrieved 319 MAGs from soil samples collected during the Brazilian Antarctic Expeditions (OPERANTAR XXXIII, XXXIV, and XXXVI) on Deception (Whalers Bay, Crater Lake, and Fumarole Bay) and Livingston (Hannah Point) Islands. On Deception Island, samples were taken along transects at Whalers Bay (33 m, four points near a thaw lake), Fumarole Bay (12 m, two points; 29.9–48.9°C soils), and Crater Lake (one point). On Livingston Island, sampling was conducted along a 27 m transect extending from the beach inland (Fig. 1).

Soil samples were collected in sterile sampling bags (~100 g per site), and genomic DNA was extracted from 0.5 g of soil using Qiagen kits according to the manufacturer’s instructions: the DNeasy PowerSoil Kit (Whalers Bay, Hannah Point) and the DNeasy PowerMax Soil Kit (Crater Lake, Fumarole Bay) (Table 1), with additional purification using the OneStep PCR Inhibitor Removal Kit (Zymo Research, USA). DNA integrity was checked on 1% agarose gels, and quantity and purity were measured using a Qubit 2.0 Fluorometer and NanoDrop ND-2000 (Thermo Fisher Scientific, USA). Whalers Bay libraries were prepared using the Illumina DNA Prep Kit and sequenced on an Illumina NovaSeq 6000 (2 × 150 bp; GenOne Biotechnologies, Brazil; ~10 M reads/sample). Libraries from Crater Lake, Fumarole Bay, and Hannah Point were prepared with the Illumina Nextera Flex DNA Library Prep Kit and sequenced on a HiSeq 2500 (2 × 100 bp; Functional Genomics Center, ESALQ/USP, Brazil; ~25 M reads/sample). Site descriptions, coordinates, and metadata are available in Supplementary data file 1 of the Zenodo data package (https://doi.org/10.5281/zenodo.14674625).

**TABLE 1:** Metagenomic sequencing data^*a*^

Site	Run accession	Sample ID	DNA extraction kit
Crater Lake	ERR15419861	CL1_2017_1	DNeasy PowerMax Soil
	ERR15419862	CL1_2017_2	
	ERR15419863	CL1_2017_3	
Fumarole Bay	ERR15419866	FU2_2017_1	DNeasy PowerMax Soil
	ERR15419867	FU2_2017_2	
	ERR15419864	FU1_2017_1	
	ERR15419865	FU1_2017_2	
Whalers Bay	ERR15419877	WB1_2014_1	DNeasy PowerSoil
	ERR15419881	WB1_2015_3	
	ERR15419882	WB1_2017_1	
	ERR15419886	WB2_2014_2	
	ERR15419887	WB2_2015_1	
	ERR15419889	WB2_2015_3	
	ERR15419891	WB2_2017_2	
	ERR15419899	WB2_2017_2	
	ERR15419900	WB3_2017_3	
	ERR15419902	WB4_2014_2	
	ERR15419884	WB1_2017_3	
	ERR15419888	WB2_2015_2	
	ERR15419894	WB3_2014_2	
	ERR15419897	WB3_2015_3	
	ERR15419898	WB3_2017_1	
	ERR15419901	WB4_2014_1	
	ERR15419903	WB4_2017_1	
	ERR15419878	WB1_2014_2	
	ERR15419879	WB1_2015_1	
	ERR15419880	WB1_2015_2	
	ERR15419883	WB1_2017_2	
	ERR15419885	WB2_2014_1	
	ERR15419890	WB2_2017_1	
	ERR15419892	WB2_2017_3	
	ERR15419893	WB3_2015_1	
	ERR15419895	WB3_2015_1	
	ERR15419896	WB3_2015_2	
	ERR15419904	WB4_2017_2	
	ERR15419905	WB4_2017_3	
Hannah Point	ERR15419868	LIV1_2017_1	DNeasy PowerSoil
	ERR15419869	LIV1_2017_2	
	ERR15419874	LIV3_2017_1	
	ERR15419876	LIV3_2017_3	
	ERR15419870	LIV1_2017_3	
	ERR15419871	LIV2_2017_1	
	ERR15419872	LIV2_2017_2	
	ERR15419873	LIV2_2017_3	
	ERR15419875	LIV3_2017_2	

^*a*^ Sampling sites on Deception (Crater Lake, Fumarole Bay, and Whalers Bay) and Livingston Islands (Hannah Point) are linked to their respective Run accession numbers (ERR accessions), the corresponding sample IDs, and the DNA extraction kit used.

Raw reads from Crater Lake, Fumarole Bay, and Hannah Point were quality-checked with FastQC v0.12.1 and trimmed with Trimmomatic v0.39 (4; Phred ≤30). High-quality reads were assembled with metaSPAdes v3.15.5 (5), and coverage was estimated via Bowtie2 v2.2.6 (6) and Samtools v0.1.19 (7). MAGs were reconstructed with Concoct v1.1 (8), MaxBin2 v2.2.6 (9), SemiBin2 v2.0.2 (10), Metacoag v1.2.0 (11), and MetaBat2 v2.12 (12), then dereplicated with MAGScoT (13). Whalers Bay data sets, previously processed (14), were assembled with MegaHit v1.2.4 (15) and binned using BinSanity v0.3.4-0 (16), MetaBAT1 v0.32.5 (17), MaxBin2 v2.2.6 (9), and Concoct v1.1 (8). Resulting bins were refined with DAS Tool v1.1.2-0 (18) and filtered with MAGpurify v2.1.2 (19), then incorporated here (Fig. 1). MAG quality was assessed with CheckM v1.1.2 (20), retaining genomes with ≥50% completeness and ≤10% contamination. Genes were predicted with Prodigal v2.6.3 (21) and taxonomy assigned using GTDB-Tk release 207 (22). All tools were run with default parameters unless otherwise specified.

The MAGs averaged 3.349 Mb and 53.88% GC, with a median completeness of 90.42% and contamination of 3.94% (Supplementary data file 3 in the Zenodo data package https://doi.org/10.5281/zenodo.14674625), and each contained ~3,350 protein-coding genes. The 319 MAGs spanned 19 bacterial phyla, including Pseudomonadota, Actinomycetota, Bacillota, and less-studied Krumholzibacteriota, Sumerleaota, and Patescibacteria, and four archaeal MAGs (three Nanoarchaeota, one Thermoproteota) (Supplementary data file 4 in the Zenodo data package https://doi.org/10.5281/zenodo.14674625). These genomes expand the Antarctic microbial genome catalog, providing new insights into microbial adaptation, ecosystem functioning, and potential responses to climate change. The study also presents a streamlined bioinformatic workflow for MAG recovery, combining multiple binning tools with refinement steps to improve genome completeness and quality. This multi-binning approach offers a practical framework for metagenomic analyses, especially in complex or low-biomass environments.

## Data Availability

All raw sequencing data and assembled genomes are publicly available in the European Nucleotide Archive (ENA) under the project accession number PRJEB95143. Individual accession numbers for the publicly available raw data are detailed in Table 1. Supplementary materials associated with this publication are accessible through the Zenodo repository (https://doi.org/10.5281/zenodo.14674625).
